# A Research Strategy to Discover the Environmental Causes of Autism and Neurodevelopmental Disabilities

**DOI:** 10.1289/ehp.1104285

**Published:** 2012-07-02

**Authors:** Philip J. Landrigan, Luca Lambertini, Linda S. Birnbaum

**Affiliations:** Philip J. Landrigan, the Ethel H. Wise Professor of Preventive Medicine, is a pediatrician and epidemiologist. He has been a member of the faculty of Mount Sinai School of Medicine since 1985, chair of the Department of Preventive Medicine since 1990, and Dean for Global Health since 2010. He is also the Director of the Children’s Environmental Health Center. He served at the Centers for Disease Control and Prevention and the National Institute for Occupational Safety and Health; he received the Meritorious Service Medal of the U.S. Public Health Service, and he was elected to the Institute of Medicine. He has published > 500 scientific papers and five books. Landrigan’s studies on the effects of low-level lead exposure in children were important in persuading the government to remove lead from gasoline and paint. He has been a leader in creating the National Children’s Study and has been involved in many studies that followed the World Trade Center Disaster on 11 September 2001.; Luca Lambertini, a molecular biologist and assistant professor of preventive medicine at the Mount Sinai School of Medicine, received his PhD from the University of Bologna in 1995. He completed postdoctoral fellowships in molecular biology at the University of Bologna and at the NIEHS. From 2004 to 2006, He was a member of the Ramazzini Institute in Bologna, where he launched a new molecular oncology program focused on exploring the cellular, genetic, and epigenetic basis for chemical carcinogenesis. His main areas of investigation have included studies of genomic imprinting, long non-protein-coding RNAs, and mitochondrial DNA methylation, and he has conducted these investigations in placental tissue to investigate genomic imprinting in the placenta in response to environmental exposures.; Linda S. Birnbaum, director of the NIEHS and the NTP, over-sees a budget that funds multidisciplinary biomedical research programs and prevention and intervention efforts that encompass training, educa-tion, technology transfer, and community outreach. She recently received an honorary Doctor of Science from the University of Rochester, the distinguished alumna award from the University of Illinois, and was elected to the Institute of Medicine. She is the author of > 700 peer-reviewed publica-tions, book chapters, abstracts, and reports. Birnbaum received her M.S. and Ph.D. in microbiology from the University of Illinois, Urbana. A board-certified toxicologist, she has served as a federal scientist for 31 years, 19 with the U.S. EPA Office of Research and Development, preceded by 10 years at the NIEHS as a senior staff fellow, a principal investigator, a research micro-biologist, and a group leader for the institute’s Chemical Disposition Group.

Autism, attention deficit/hyperactivity disorder (ADHD), mental retardation, dyslexia, and other biologically based disorders of brain development affect between 400,000 and 600,000 of the 4 million children born in the United States each year. The Centers for Disease Control and Prevention (CDC) has reported that autism spectrum disorder (ASD) now affects 1.13% (1 of 88) of American children (CDC 2012) and ADHD affects 14% (CDC 2005; Pastor and Reuben 2008). Treatment of these disorders is difficult; the disabilities they cause can last lifelong, and they are devastating to families. In addition, these disorders place enormous economic burdens on society (Trasande and Liu 2011).

Although discovery research to identify the potentially preventable causes of neuro-develop-mental disorders (NDDs) has increased in recent years, more research is urgently needed. This research encompasses both genetic and environ-mental studies.

Genetic research has received particular investment and attention (Autism Genome Project Consortium et al. 2007; Buxbaum and Hof 2011; Fernandez et al. 2012; O’Roak et al. 2011; Sakurai et al. 2011) and has demonstrated that ASD and certain other NDDs have a strong hereditary component (Buxbaum and Hof 2011; Sakurai et al. 2011). Linkage studies have identified candidate autism susceptibility genes at multiple loci, most consistently on chromosomes 7q, 15q, and 16p (Autism Genome Project Consortium et al. 2007; Sakurai et al. 2011). Exome sequencing in sporadic cases of autism has detected new mutations (O’Roak et al. 2011), and copy number variant studies have identified several hundred copy number variants putatively linked to autism (Fernandez et al. 2012). The candidate genes most strongly implicated in NDD causation encode for proteins involved in synaptic architecture, neuro-transmitter synthesis (e.g., ©-amino-butyric acid serotonin), oxytocin receptors, and cation trafficking (Sakurai et al. 2011). No single anomaly predominates. Instead, autism appears to be a family of diseases with common pheno-types linked to a series of genetic anomalies, each of which is responsible for no more than 2–3% of cases. The total fraction of ASD attributable to genetic inheri-tance may be about 30–40%.

Exploration of the environmental causes of autism and other NDDs has been catalyzed by growing recognition of the exquisite sensitivity of the developing human brain to toxic chemicals (Grandjean and Landrigan 2006). This susceptibility is greatest during unique “windows of vulnerability” that open only in embryonic and fetal life and have no later counter-part (Miodovnik 2011). “Proof of the principle” that early exposures can cause autism comes from studies linking ASD to medications taken in the first trimester of pregnancy—thalidomide, misoprostol, and valproic acid—and to first-trimester rubella infection (Arndt et al. 2005; Daniels 2006).

This “proof-of-principle” evidence for environmental causation is supported further by findings from prospective birth cohort epidemio-logical studies, many of them supported by the National Institute of Environmental Health Sciences (NIEHS). These studies enroll women during pregnancy, measure prenatal exposures in real time as they occur, and then follow children longitudinally with periodic direct examinations to assess growth, development, and the presence of disease. Prospective studies are powerful engines for the discovery of etiologic associations between prenatal exposures and NDDs. They have linked autistic behaviors with prenatal exposures to the organophosphate insecticide chlorpyrifos (Eskenazi et al. 2007) and also with prenatal exposures to phthalates (Miodovnik et al. 2011). Additional prospective studies have linked loss of cognition (IQ), dyslexia, and ADHD to lead (Jusko et al. 2008), methyl-mercury (Oken et al. 2008), organophosphate insecticides (London et al. 2012), organo-chlorine insecticides (Eskenazi et al. 2008), polychlorinated biphenyls (Winneke 2011), arsenic (Wasserman et al. 2007), manganese (Khan et al. 2011), polycyclic aromatic hydrocarbons (Perera et al. 2009), bisphenol A (Braun et al. 2011), brominated flame retardants (Herbstman et al. 2010), and perfluorinated compounds (Stein and Savitz 2011).

Toxic chemicals likely cause injury to the developing human brain either through direct toxicity or inter-actions with the genome. An expert committee convened by the U.S. National Academy of Sciences (NAS) estimated that 3% of neuro-behavioral disorders are caused directly by toxic environ-mental exposures and that another 25% are caused by inter-actions between environmental factors, defined broadly, and inherited susceptibilities (National Research Council 2000). Epigenetic modification of gene expression by toxic chemicals that results in DNA methyla-tion, histone modification, or changes in activity levels of non-protein-coding RNA (ncRNAs) may be a mechanism of such gene–environment interaction (Grafodatskaya et al. 2010). Epigenetic “marks” have been shown to be able to influence gene expression and alter high-order DNA structure (Anway and Skinner 2006; Waterland and Jirtle 2004).

A major unanswered question is whether there are still undiscovered environ-mental causes of autism or other NDDs among the thousands of chemicals currently in wide use in the United States. In the past 50 years, > 80,000 new synthetic chemicals have been developed (Landrigan and Goldman 2011). The U.S. Environmental Protection Agency has identified 3,000 “high production volume” (HPV) chemicals that are in widest use and thus pose greatest potential for human exposure (Goldman 1998). These HPV chemicals are used today in millions of consumer products. Children and pregnant women are exposed extensively to them, and CDC surveys detect quantifiable levels of nearly 200 HPV chemicals in the bodies of virtually all Americans, including pregnant women (Woodruff et al. 2011).

The significance of early chemical exposures for children’s health is not yet fully understood. A great concern is that a large number of the chemicals in widest use have not undergone even minimal assessment of potential toxicity, and only about 20% have been screened for potential toxicity during early development (Landrigan and Goldman 2011). Unless studies specifically examine develop-mental consequences of early exposures to untested chemicals, sub-clinical dysfunction caused by these exposures can go unrecognized for years. One example is the “silent epidemic” of childhood lead poisoning: From the 1940s to the 1980s, millions of American children were exposed to excessive levels of lead from paint and gasoline, resulting in reduced average intelligence by 2–5 IQ points (Grosse et al. 2002). The late David Rall, former director of NIEHS, once observed that “If thalidomide had caused a 10-point loss of IQ instead of birth defects of the limbs, it would likely still be on the market” (Weiss 1982).

To begin formulation of a systematic strategy for discovery of potentially preventable environmental causes of autism and other NDDs, the Mount Sinai Children’s Environmental Health Center, with the support of the NIEHS and Autism Speaks, convened a workshop on “Exploring the Environmental Causes of Autism and Learning Disabilities.” This workshop produced a series of papers by leading researchers, some of which are published in this issue of *Environmental Health Perspectives*. It also generated a list of 10 chemi-cals and mixtures widely distributed in the environment that are already suspected of causing develop-mental neuro-toxicity:

Lead (Jusko et al. 2008)
Methylmercury (Oken et al. 2008)
Polychlorinated biphenyls (Winneke 2011)
Organophosphate pesticides (Eskenazi et al. 2007; London et al. 2012)
Organochlorine pesticides (Eskenazi et al. 2008)
Endocrine disruptors (Braun et al. 2011; Miodovnik et al. 2011)
Automotive exhaust (Volk et al. 2011)
Polycyclic aromatic hydrocarbons (Perera et al. 2009)
Brominated flame retardants (Herbstman et al. 2010)
Perfluorinated compounds (Stein and Savitz 2011).

This list is not exhaustive and will almost certainly expand in the years ahead as new science emerges. It is intended to focus research in environmental causation of NDDs on a short list of chemicals where concentrated study has high potential to generate actionable findings in the near future. Its ultimate purpose is to catalyze new evidence-based programs for prevention of disease in America’s children.
